# The effect of chronic regulatory focus and social comparison on undergraduates’ intertemporal choices under gain-loss frame

**DOI:** 10.3389/fpsyg.2022.1076304

**Published:** 2023-01-06

**Authors:** Dan Liu, Yuan Zhang, Xiaowei Geng

**Affiliations:** ^1^School of Educational Science, Ludong University, Yantai, China; ^2^Institute for Education and Treatment of Problematic Youth, Ludong University, Yantai, China; ^3^Jing Hengyi School of Education, Hangzhou Normal University, Hangzhou, China

**Keywords:** social comparison, the chronic regulatory focus, gain-loss frames, intertemporal choice, undergraduate

## Abstract

Intertemporal choice refers to decisions involving tradeoffs among costs and benefits occurred at different times. To investigate whether college students’ intertemporal decision making under the gain and loss frames is affected by their chronic regulatory focus. Currently, experiment 1 investigated the influence of college students’ chronic regulatory focus on intertemporal decision making under the gain and loss frames, and experiment 2 further explored the moderating effect of social comparison (i.e., upward or downward social comparison) during this process. The results showed that intertemporal choices of participants with promotive focus was no significant difference between the gain frame and loss frame, while college students with preventive focus chose later and larger rewards more in loss frame than in gain frame. Social comparison moderated the effects of the chronic regulatory focus on college students’ intertemporal choice in gain and loss frames. The upward comparison enhanced the influence of regulatory focus on intertemporal choice in the gain and loss frames, while the downward comparison weakened it.

## 1. Introduction

Intertemporal decision making refers to a decision that involves tradeoffs in costs and benefits occurring at different times (Frederick et al., 2002; Ren et al., 2015). In daily life, sometimes people make choices in order to obtain gains, and sometimes to avoid losses. When the same content is expressed in different frames, individual’s choice in the same task will be affected, the decision preference can even be reversed, which is called Framing Effect (Tversky and Kahneman, 1981). Individuals’ intertemporal choice are also different in the gains and losses (Gehring and Willoughby, 2002; Xu et al., 2009). Specifically, compared to gains, individuals in the losses take a more long-term perspective and are more inclined to a later-larger benefit (LL; Thaler, 1981). Previous studies have further confirmed the existence of gain and loss framing effect in intertemporal decision making (Ma et al., 2012). Participants were more likely to choose sooner-smaller benefit (SS) under the gain frame than under the loss frame (Wu et al., 2016). These conclusions were also supported by recent electrophysiological studies, which showed that different cognitive responses were elicited when making decisions under gain-loss frames (Zhang, 2018).

In general, previous studies mainly focused on the differences in intertemporal decision making under the gain-loss frames and its neural mechanisms. The influence of personal factors such as age (Zilker and Pachur, 2021) and self-control (Faralla et al., 2017) were also investigated as well, but few studies have examined the effect of chronic regulatory focus on intertemporal decision making under the gain-loss frames.

In recent years, undergraduates have many problems on money choices, such as the problem of borrowing, living expenses over value, which are related with their intertemporal choices. Therefore, it is important to investigate college students’ intertemporal choices. The present research aims to address this issue by examining the effect of chronic regulatory focus on undergraduates’ intertemporal choices in gain/loss frame and the moderation of social comparison.

Regulatory Focus Theory (RFT) refers to the way which people constantly self-regulate in order to achieve a desired goal (Higgins, 1997), and individuals’ stable personality traits during self-regulation is called chronic regulatory focus (Wang et al., 2011). Studies have confirmed that chronic regulatory focus plays an important role in making judgments and decisions (Pham and Higgins, 2005). Individuals with different regulatory focus respond differently to positive and negative outcomes, promotive focus participants responding more sharply to gains and preventive focus participants behaving more sharply to losses (Werth and Foerster, 2007; Novak and Hoffman, 2009; Yao and Yue, 2009). Previous studies have also investigated the relationship between regulatory focus and intertemporal choices. However, the findings were not consistent. For example, Wang et al. (2019) pointed out that participants with a promotive focus preferred more immediate rewards, and participants with a preventive focus preferred delayed rewards. Zhou and Zhao (2009) found that compared with preventive focus people, promotive focus individuals have a lower discount rate on gains and a higher discount rate on losses. In the study of Zhou and Zhao (2009), to measure the delay discounting rate in intertemporal choices, they only used two filling blank questions (one in the gain domain and the other in the loss domain), i.e., matching method. Previous studies found that the matching method may not be conducive to the presentation of relatively complex situations (David et al., 2013; Chen and He, 2015). Thus, more evidences on the relationship between regulatory focus and intertemporal choices in gain/loss frame are needed.

According to Regulatory Focus Theory (RFT), individuals with promotive focus actively strive for achievement goals they wanted, while individuals with preventive focus, driven by safety needs, do their best to fulfill their responsibilities and obligations as well as avoid losses. Therefore, gains-loss situations may also have a different impact on individuals with different chronic regulatory focus. Due to the stronger motivation to obtain greater gains individuals with promotive focus will prefer to choose LL in gain situations, while preventive focus individuals who have stronger motivation to avoid larger losses tend to choose later-smaller losses (i.e., *later-larger benefit*) in loss situations. Thus, the hypothesis is as followed,

*H1*: Chronic regulatory focus influences intertemporal choices in the gain-loss frames. Undergraduates with promotive focus prefer later-larger benefit more in gain frame than in loss frame. And those who with preventive focus prefer later-smaller losses (i.e., later-larger benefit) more in loss frame than in gain frame.

Social Comparison Theory (SCT) proposed that individuals need to evaluate their own views through objective information, so information from others as data source is selected to judge their own situation (Festinger, 1954). Social comparison refers to the tendency of human beings to compare their abilities, wealth and social status with others in order to draw complete pictures of themselves (Dou et al., 2014). Previous studies showed that taking others as reference can influence individuals’ decision making. For example, when participants observe farsighted others, they tend to choose later and larger choices; when participants observe shortsighted others, they tend to choose small and sooner choices (Gilman et al., 2014; Calluso et al., 2017).

Social comparison can be divided into upward and downward social comparison depending on the direction of comparison. Upward social comparison is the process of comparing with others who are better than oneself, while downward is the process of comparing with the worse (Wills, 1981; Lian et al., 2017; Liu et al., 2019; Vogel et al., 2020). Previous studies suggested that upward social comparison could be threatening for individuals’ self-concepts (Pettit and Lount, 2010). When comparing with those who are better than them, i.e., upward comparison, one may feel ego-threatened (Han and Chi, 2012), i.e., a threat to a person’s self-image or self-esteem. People often make some changes with a compensatory purpose under upward comparison (Gong and Zhang, 2020). Thus, when compared upward, promotive focus individuals will try hard to eliminate the ego-threat through positive ways like gaining more money and achieving good reputations based on its sensitivity of gains. Thus, they tend to prefer LL choice to a greater extent in gain frames than loss frames. Similarly, preventive focus individuals may feel ego-threatened when compared upward. Preventive focus individuals are more sensitive to losses, and will prefer LL to a greater extent in the loss frame than gain frame when upward social comparisons are made. In contrary, downward social comparison is the way people compare themselves with individuals who are inferior to them, so they feel better about themselves (Zheng et al., 2015), which may bring positive emotions to individuals, reduce stress and increase self-esteem (Xing and Yu, 2006; Guo and Huang, 2010). In downward social comparison, people experienced better self-evaluation and relatively higher self-esteem (Han and Chi, 2012). Previous studies found that self-esteem was related with people’s impulsive buying (Dhandra, 2020). Therefore, the experience of success compared with others makes the promotive focus and preventive focus individuals have no strong desire to change current status. In other words, under downward social comparison, the motivations of promotive focus individuals to gain more and preventive focus individuals to avoid greater losses would be both weakened. In short, the difference in intertemporal decision making between promotive-focus and preventive-focus people under gain-loss frames becomes smaller. Therefore, we came up with the following hypothesis,

*H2*: Upward social comparison elevates the effect of chronic regulatory focus on intertemporal decision making in gain-loss frames, while downward social comparison attenuates this effect.

Two experiments were conducted to test the two hypotheses. Experiment 1 examined the effect of chronic regulatory focus on intertemporal decision making in gain-loss frames. Experiment 2 further explored the moderating role of social comparison in this process.

## 2. Experiment 1: The effect of chronic regulatory focus on intertemporal choices in gain and loss frames

### 2.1. Participants

A total of 83 undergraduates were recruited from a university, including 16 males (*M* = 19.75, *SD* = 1.39) and 67 females (*M* = 19.34, *SD* = 1.34). According to G*Power 3.1 (Faul et al., 2007), under the premise of statistical test force 1–*β* = 0.8, bilateral test *ɑ* = 0.05, and effect size *f* = 0.25, the number of subjects for repeated measures ANOVA should be set to 66. In this study, there was no significant difference in intertemporal decision making between males (*M* = −5.94, *SD* = 2.21) and females (*M* = −6.26, *SD* = 1.45) in the loss frame, *t*(81) = 0.72, *p* = 0.473, *p* > 0.05, 95% CI = [−0.57, 1.22] and no significant difference in intertemporal decision making between males (*M* = −5.43, *SD* = 1.97) and females (*M* = −5.82, *SD* = 1.31) in the gain frame, *t*(81) = 0.97, *p* = 0.335, *p* > 0.05, 95% CI = [−0.41, 1.20].

### 2.2. Design

A 2 (chronic regulatory focus: promotive focus vs. preventive focus) × 2 (gain-loss frames: gain vs. loss) design was used. The chronic regulatory focus was a between-subject factor and the gain-loss frames was a within-subject factor. Forty-four participants were promotive focus, 39 participants were preventive focus. The dependent variable was the discounting rate of intertemporal decision making.

### 2.3. Materials

1. Chronic regulatory focus questionnaire. We used the Regulatory Focus Questionnaire (Chinese version), revised by Yao and Yue (2009). The questionnaire includes 10 items, 6 questions for promotive focus (1, 3, 7, 8, 9, 10, in which 1, 8, 10 questions were reversed scored); 4 questions for preventive focus (2, 4, 5, 6, in which 2, 4, 6 questions were reversed scored). Participants reported their opinions on a 5-point scale ranging from “1 (totally disagree)” to “5 (totally agree).” By calculating the subjects’ scores on the two subscales of promotive focus and preventive focus, the difference value was transformed into a Z-score. According to previous research (Yao et al., 2005), participants with positive Z-score was coded as promotive focus, and those with negative Z-score was coded as preventive focus. In previous research (Yao et al., 2005), the reliability coefficient Cronbach’s *α* = 0.73. In the present study, Cronbach’s *α* = 0.65.
2. Intertemporal choices in the gain-loss frames were measured with a well-validated and widely used monetary choice questionnaire with 27 items (MCQ; Kirby et al., 1999; Kirby, 2009). All items were choice questions. As the original questionnaire was in US dollars, the values in the original questionnaire were multiplied by 7 (the average of the exchange rates) to make them suitable for Chinese people. The details are as follow.

Gain frame: Assuming that you were paid ¥700 for this experiment as a participant. However, because you are required to pay a certain amount of tax according to experimenter’s agreement, you actually receive less than ¥700. Now we offer you two options to get your payment. For example: “Receive ¥140 today” or “Receive ¥385 after 7 days.”

Loss frame: Assuming that you, as a participant in this experiment, should get ¥700 as experimental payment. However, due to your mistake, the data is invalid. According to the experimenter agreement filled in before, part of the amount will be deducted and you actually receive less than ¥700. Now we offer you two options of reparations. For example: “Pay ¥560 today” or “Pay ¥315 after 7 days.”

Participants’ responses on each trial were converted to delay discounting rates by using equation V = 1/(A + kD), where V is the present value of the delayed reward A at delay D, and k is a free parameter that determines the discounting rate. All delays are measured in days, and the values of k are scaled accordingly, with lower values corresponding to higher levels of foresightedness (Kirby et al., 1999).

### 2.4. Procedure

**Figure 1:**
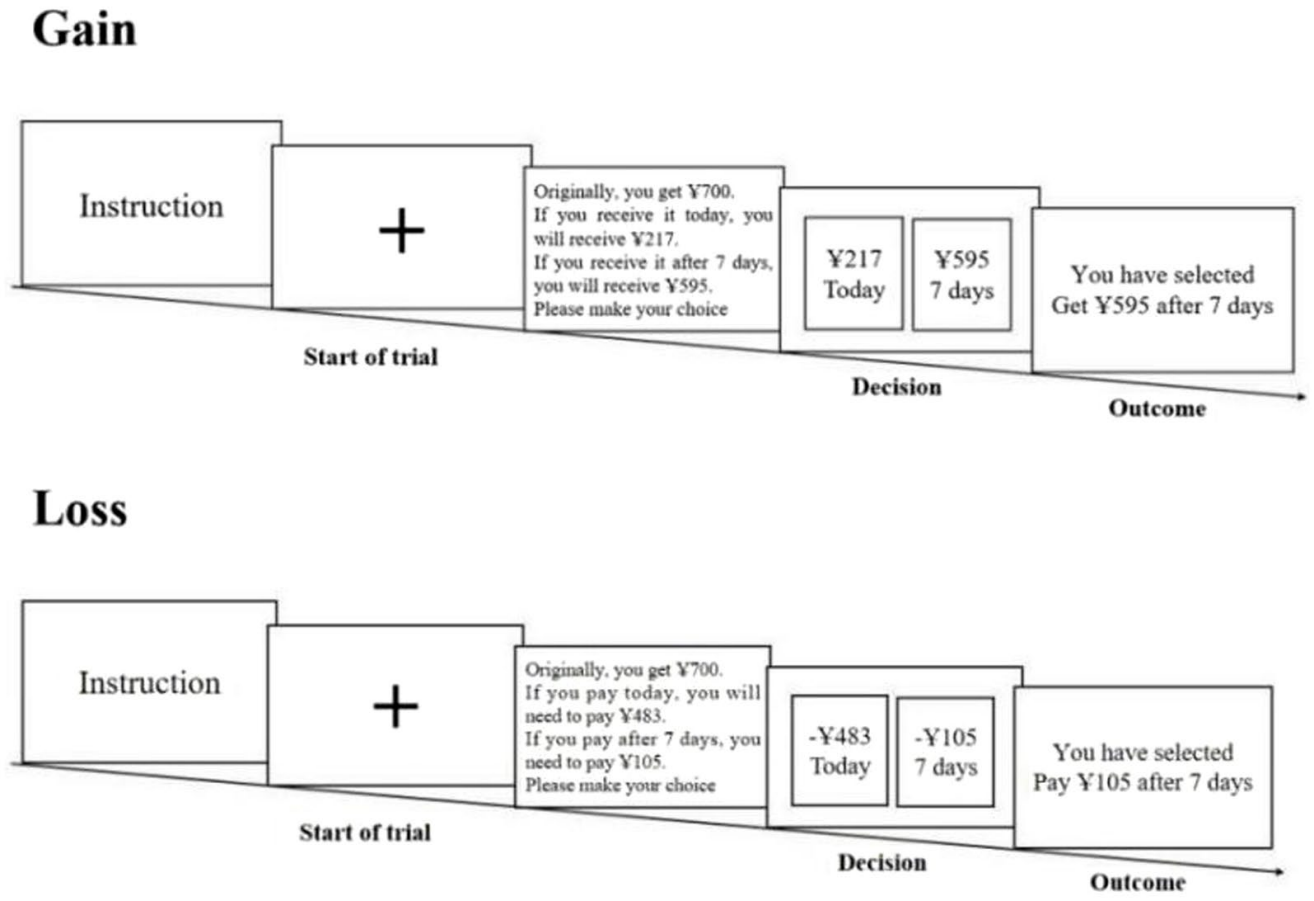
Experimental flow chart in the gain-loss frames.

First, participants were asked to complete the chronic regulatory focus questionnaire. Then, the intertemporal choices in the gain and loss frames were presented to the subjects *via* E-prime 2.0 using balanced sequence (see Figure 1 for details). According to their actual choices, they got payoff, in a certain proportion, e.g., if the participant chose to get it after 7 days, the payoff would be paid after 7 days.

### 2.5. Data analysis

First, we investigated the effect of chronic regulatory focus on intertemporal decision making under the gain-loss frames. The ANOVA results showed that the main effect of the gain-loss frames was significant *F*(1, 81) = 8.05, *p* = 0.006, *p* < 0.05, *η*_p_^2^ = 0.09, the delay discounting rate was smaller under the loss frame (*M* = −6.20, *SD* = 1.61) than under the gain frame (*M* = −5.75, *SD* = 1.46), and participants preferred later and smaller losses under the loss frame, that is to say, they preferred later and larger gains. The main effect of chronic regulation focus was not significant, *F*(1, 81) = 0.84, *p* = 0.362, *p* > 0.05, *η*_p_^2^ = 0.01; the interaction between chronic regulatory focus and intertemporal choices in the gain-loss frames was significant, *F*(1, 81) = 7.72, *p* = 0.007, *p* < 0.05, *η*_p_^2^ = 0.09.

**Figure 2:**
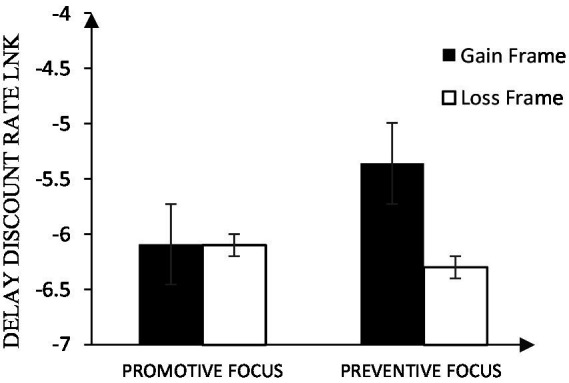
Effects of chronic regulatory focus on intertemporal decision making in the gain-loss frames.

Further analysis about the differences between gain-loss frame among regulatory focus, results showed that for promotive focus participants, there was no significant difference in delay discounting rates between the loss frame (*M* = −6.10, *SD* = 1.63) and the gain frame (*M* = −6.09, *SD* = 1.32), *t*(43) = 0.04, *p* = 0.996, *p >* 0.05, 95% CI = [−0.46, 0.48], Cohen’s *d* = 0.01. For preventive focus participants, delay discounting rates were smaller in the loss frame (*M* = −6.30, *SD* = 1.61) compared to that in the gain frame (*M* = −5.36, *SD* = 1.51), *t*(38) = 3.87, *p <* 0.001, 95% CI = [0.45, 1.44], Cohen’s *d* = 0.60, see Figure 2; Table 1.

**Table 1:** An paired sample *t*-test for intertemporal decision making between promotive focus and preventive focus.

Chronic regulatory focus	Measure	Intertemporal decision making	*t*	*df*	*p*	95% CI	*d*
Promotive focus	Gain frame	−6.09 ± 1.32	0.04	43	0.966	[−0.46, 0.48]	0.01
	Loss frame	−6.10 ± 1.63					
Preventive focus	Gain frame	−5.36 ± 1.51	3.87	38	<0.001	[0.45, 1.44]	0.60
	Loss frame	−6.30 ± 1.61					

And results about the differences between promotive and preventive focus among gain-loss frame showed that, in the gain frame, delay discounting rates were smaller with promotive focus participants (*M* = −6.09, *SD* = 1.32) compared to that with preventive focus participants (*M* = −5.36, *SD* = 1.51), *t*(81) = −2.35, *p* = 0.021, *p* < 0.05, 95% CI = [−1.35, −0.11], Cohen’s *d* = −0.51. In the loss frame, there was no significant difference in delay discounting rates between promotive focus (*M* = −6.10, *SD* = 1.63) and preventive focus (*M* = −6.30, *SD* = 1.61), *t*(81) = 0.57, *p* = 0.570, *p >* 0.05, 95% CI = [−0.50, 0.91], Cohen’s *d* = 0.79, see Table 2.

**Table 2:** An independent sample *t*-test for intertemporal decision making between promotive focus and preventive focus.

Measure	Chronic regulatory focus	Intertemporal decision making	*t*	*df*	*p*	95% CI	*d*
Gain frame	Promotive focus	−6.09 ± 1.32	−2.35	81	0.021	[−1.35, -0.11]	−0.51
	Preventive focus	−5.36 ± 1.51					
Loss frame	Promotive focus	−6.10 ± 1.63	0.57	81	0.570	[−0.50, 0.91]	0.79
	Preventive focus	−6.30 ± 1.61					

### 2.6. Experiment 1 results

Experiment 1 found that chronic regulatory focus influenced intertemporal decision making in the gain-loss frames, which was consistent with the hypothesis. Participants with promotive focus did not show significant difference in intertemporal choices under the gain and loss frame. Delay discounting rates of participants with preventive focus were smaller under the loss frame than that under the gain frame. In the gain frame, participants with promotive focus were more inclined to choose smaller delay discounting rates than participants with preventive focus. In the loss frame, participants with preventive focus and promotive focus did not show significant difference in intertemporal choices.

Experiment 2 would build on experiment I by further examining the role of social comparison in the influence of chronic regulatory focus on intertemporal decision making in the gain-loss frames.

## 3. Experiment 2: The moderating role of social comparison in the influence of chronic regulatory focus on intertemporal choices in gain and loss frames

### 3.1. Participants

A total of 92 undergraduates were recruited from a university, including 26 males (*M* = 19.92, *SD* = 1.47) and 66 females (*M* = 19.06, *SD* = 1.01). According to G*Power 3.1 (Faul et al., 2007), under the premise of statistical test force 1–*β* = 0.8, bilateral test *ɑ* = 0.05, and effect size *f* = 0.25, the number of subjects for repeated measures ANOVA should be set to 92.

In this study, there was no significant difference in intertemporal decision making between males (*M* = −5.39, *SD* = 2.46) and females (*M* = −5.90, *SD* = 2.48) in the loss frame, *t*(90) = 0.89, *p* = 0.374, *p* > 0.05, 95% CI = [−0.63, 1.65], and no significant difference in intertemporal decision making between males (*M* = −4.17, *SD* = 1.94) and females (*M* = −4.78, *SD* = 2.03) in the gain frame, *t*(90) = 1.31, *p* = 0.192, *p* > 0.05, 95% CI = [−0.31, 1.53].

### 3.2. Design

A 2 (chronic regulatory focus: promotive focus vs. preventive focus) × 2 (social comparison: upward vs. downward social comparison) × 2 (gain-loss frames: gain vs. loss) design was used. The chronic regulatory focus and social comparison were between-subjects factors and the gain-loss frames was a within-subject factor. The dependent variable was delay discounting rates of intertemporal choices.

### 3.3. Materials

1. Chronic regulatory focus questionnaire, same as experiment 1.
2. Intertemporal choices in the gain-loss frames, same as experiment 1.
3. Social comparison. Previous studies usually manipulate social comparison by comparing sores of one kind of test, which can be a good way to prime social comparison (Lockwood and Kunda, 1997; Zheng et al., 2015; Wang et al., 2016; Yao et al., 2022). Based on these previous studies, we designed an academic exam to evaluate participants’ math level. Participants were presented with six arithmetic questions. For example, “29 + 18 – 15 − 2 + 12 − 17 + 11 − 22 + 18 − 4 = ?.” In upward comparison condition, participants were told “Your score is in the bottom 10% of your classmates who took the test” and in downward comparison condition, participants were told “Your score is in the top 10% of your classmates who took the test.” Each participant was clearly informed about the rank. After the feedback, participants were asked to evaluate their performance in the math test, for example, “How do you feel about your math skills?,” “Do you think you did well on this math test?.” In addition, to exclude the effect of social comparison on emotions, six questions (e.g., excited, upset) were asked to measure positive and negative emotions (Zheng et al., 2015).

To check the effectiveness of social comparison priming, a total of 114 undergraduates were recruited from a university, including 30 males (*M* = 20.43, *SD* = 1.28) and 84 females (*M* = 20.44, *SD* = 1.47). Social comparison had no significant effect on individual mental arithmetic level (“How do you feel about your mental math skills?”) *F*(1, 112) = 0.01, *p* = 0.926, *p* > 0.05, *η*_p_^2^ = 0.01. Social comparison significantly influenced the level of performance on this test (“Do you think you did well on this mental math test?”), *F*(1, 112) = 6.21, *p* = 0.014, *p* < 0.05, *η*_p_^2^ = 0.53. Participants with downward social comparison (*M* = 3.76, *SD* = 1.13) were more satisfied with their performance on this test than those with upward social comparison (*M* = 3.16, *SD* = 1.42). One-way ANOVA showed that social comparison had no significant effect on participants’ positive emotional experience, *F*(1, 112) = 3.63, *p* = 0.059, *p* > 0.05, *η*_p_^2^ = 0.03 and negative emotional experiences, *F*(1, 112) = 0.64, *p* = 0.426, *p* > 0.05, *η*_p_^2^ = 0.01. This was consistent with results of Zheng et al. (2015) and which showed that the manipulation of social comparison was effective.

### 3.4. Procedure

First, participants were asked to complete the chronic regulatory focus questionnaire. Then, they were randomly assigned into upward social comparison or downward social comparison *via* E-prime 2.0. Finally, participants were presented with intertemporal choices items under the gain and loss frames, which was the same as in experiment 1. All participants were paid after experiment.

### 3.5. Data analysis

**Figure 3:**
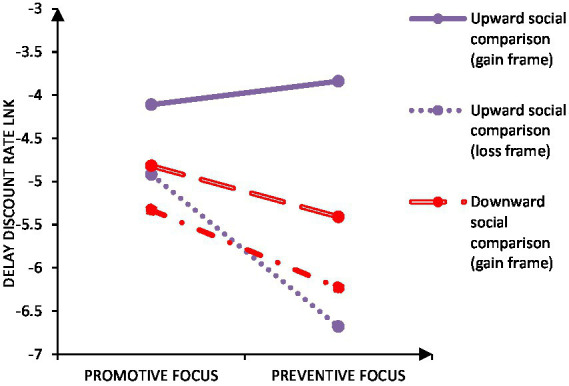
Effects of social comparison and chronic regulatory focus on intertemporal decision making in the gain-loss frames.

First, we investigated the effect of chronic regulatory focus and social comparison on intertemporal choices under the gain-loss frames, *F*(1, 88) = 50.21, *p* < 0.001, *η*_p_^2^ = 0.36. The delay discounting rate was smaller in the loss frame (*M* = −5.79, *SD* = 0.25) than in the gain frame (*M* = −4.55, *SD* = 0.21). The main effect of social comparison was not significant, *F*(1, 88) = 1.72, *p* = 0.193, *p* > 0.05, *η*_p_^2^ = 0.02. The main effect of chronic regulatory focus was not significant *F*(1, 88) = 3.02, *p* = 0.086, *p* > 0.05, *η*_p_^2^ = 0.03. The interaction of chronic regulatory focus and social comparison was not significant, *F*(1, 88) = 0.01, *p* = 0.994, *p* > 0.05, *η*_p_^2^ = 0.01. The interaction of gain-loss frames and social comparison was significant, *F*(1, 88) = 10.87, *p* = 0.001, *p* < 0.05, *η*_p_^2^ = 0.11. The interaction of gain-loss frames and chronic regulatory focus was significant, *F*(1, 88) = 11.17, *p* = 0.001, *p* < 0.05, *η*_p_^2^ = 0.11. The interaction of social comparison, chronic regulatory focus, and gain-loss frames three variables was significant, *F*(1, 88) = 5.94, *p* = 0.017, *p* < 0.05, *η*_p_^2^ = 0.06, see Figure 3.

The interaction of gain-loss frames and social comparison was significant. Further analysis about the differences between gain-loss frame among social comparison, results showed that for upward social comparison, delay discounting rates were smaller in the loss frame (*M* = −5.74, *SD* = 2.45) compared to that in the gain frame (*M* = −3.99, *SD* = 1.96), *t*(40) = 5.82, *p* > 0.001, 95% CI = [1.14, 2.36], Cohen’s *d* = 0.79. For downward social comparison, delay discounting rates were smaller in the loss frame (*M* = −5.77, *SD* = 2.51) compared to that in the gain frame (*M* = −5.11, *SD* = 1.93), *t*(50) = 2.82, *p* = 0.007, 95% CI = [0.19, 1.14], Cohen’s *d* = 0.29. And results about the differences between social comparison among gain-loss frame showed that, in the gain frame, delay discounting rates were smaller with downward social comparison (*M* = −5.11, *SD* = 1.93) compared to that with upward social comparison (*M* = −3.99, *SD* = 1.96), *t*(90) = −2.76, *p* = 0.007, *p* < 0.05, 95% CI = [−1.93, −0.31], Cohen’s *d* = 0.58. In the loss frame, there was no significant difference in delay discounting rates between upward social comparison (*M* = −5.74, *SD* = 2.45) and downward social comparison (*M* = −5.77, *SD* = 2.51), *t*(90) = −0.07, *p* = 0.945, *p* > 0.05, 95% CI = [−1.07, 1.00], Cohen’s *d* = 0.01.

The interaction of gain-loss frames and chronic regulatory focus was significant. Further analysis about the differences between gain-loss frame among regulatory focus, results showed that for promotive focus participants, delay discounting rates were smaller in the loss frame (*M* = −5.15, *SD* = 2.55) compared to that in the gain frame (*M* = −4.50, *SD* = 2.11), *t*(47) = 2.84, *p* = 0.007, *p* < 0.05, 95% CI = [0.19, 1.10], Cohen’s *d* = 0.28. For preventive focus participants, delay discounting rates were smaller in the loss frame (*M* = −6.43, *SD* = 2.22) compared to that in the gain frame (*M* = −4.73, *SD* = 1.92), *t*(43) = 5.58, *p* < 0.001, 95% CI = [1.08, 2.31], Cohen’ s *d* = 0.82, contrary to the result of the experiment 1. And results about the differences between regulatory focus among gain-loss frame showed that, in the gain frame, there was no significant difference in delay discounting rates between promotive focus (*M* = −4.50, *SD* = 2.11) and preventive focus (*M* = −4.73, *SD* = 1.92), *t*(90) = 0.55, *p* = 0.587, *p* > 0.05, 95% CI = [−6.05, 1.07], Cohen’s *d* = 0.11. In the loss frame, delay discounting rates were smaller with preventive focus participants (*M* = −6.43, *SD* = 2.22) compared to that with promotive focus participants (*M* = −5.15, *SD* = 2.55), *t*(90) = 2.56, *p* = 0.012, *p* < 0.05, 95% CI = [0.29, 2.28], Cohen’s *d* = 0.54, different from experiment 1. What makes this result different from experiment 1 is the initiation of social comparison.

In order to further analyze the interaction among social comparison, chronic regulatory focus, and gain-loss frames, we analyzed the influence of chronic regulatory focus and gain-loss frames on intertemporal decision-making under the conditions of upward and downward social comparison, respectively. In the upward social comparison condition, the main effect of gain-loss frames on delay discounting rate was significant, *F*(1, 39) = 49.81, *p* < 0.001, *η*_p_^2^ = 0.56. Delay discounting rate was smaller under the loss frame (*M* = −5.80, *SD* = 0.36) than under the gain frame (*M* = −3.98, *SD* = 0.31). The main effects of chronic regulatory focus was not significant, *F*(1, 39) = 1.43, *p* = 0.239, *p* > 0.05, *η*_p_^2^ = 0.04. The interaction of gain-loss frames and chronic regulatory focus was significant, *F*(1, 39) = 15.44, *p* < 0.001, *η*_p_^2^ = 0.28. Simple effects analysis about the differences between gain-loss frame among regulatory focus, results showed that for promotive focus participants, delay discounting rates were smaller in the loss frame (*M* = −4.92, *SD* = 2.59) compared to that in the gain frame (*M* = −4.11, *SD* = 2.18), *t*(21) = 2.55, *p* = 0.019, *p* < 0.05, 95% CI = [0.15, 1.47], Cohen’s *d* = 0.34. For preventive focus participants, delay discounting rates were smaller in the loss frame (*M* = −6.68, *SD* = 1.94) compared to that in the gain frame (*M* = −3.84, *SD* = 1.71), *t*(18) = 6.80, *p* < 0.001, 95% CI = [1.96, 3.73], Cohen’s *d* = 1.55, see Table 3. And results about the differences between regulatory focus among gain-loss frame showed that, in the gain frame, there was no significant difference in delay discounting rates between promotive focus (*M* = −4.11, *SD* = 2.18) and preventive focus (*M* = −3.84, *SD* = 1.71), *t*(39) = −0.44, *p* = 0.663, *p* > 0.05, 95% CI = [−1.53, 0.98], Cohen’s *d* = −0.14. In the loss frame, delay discounting rates were smaller with preventive focus participants (*M* = −6.68, *SD* = 1.94) compared to that with promotive focus participants (*M* = −4.92, SD = 2.59), *t*(39) = 2.43, *p* = 0.020, *p* < 0.05, 95% CI = [0.30, 3.23], Cohen’s *d* = 0.77, see Table 4.

**Table 3:** An paired sample *t*-test for intertemporal decision making between promotive focus and preventive focus in the condition of upward social comparison.

Chronic regulatory focus	Measure	Intertemporal decision making	*t*	*df*	*p*	95% CI	*d*
Promotive focus	Gain frame	−4.11 ± 2.18	2.55	21	0.019	[0.15, 1.47]	0.34
	Loss frame	−4.92 ± 2.59					
Preventive focus	Gain frame	−3.84 ± 1.71	6.80	18	<0.001	[1.96, 3.72]	1.55
	Loss frame	−6.68 ± 1.94					

**Table 4:** An independent sample *t*-test for intertemporal decision making between promotive focus and preventive focus in the condition of upward social comparison.

Measure	Chronic regulatory focus	Intertemporal decision making	*t*	*df*	*p*	95% CI	*d*
Gain frame	Promotive focus	−4.11 ± 2.18	−0.44	39	0.663	[−1.53, 0.98]	−0.14
	Preventive focus	−3.84 ± 1.71					
Loss frame	Promotive focus	−4.92 ± 2.59	2.43	39	0.020	[0.30, 3.23]	0.77
	Preventive focus	−6.68 ± 1.94					

In the downward social comparison condition, the effects of chronic regulatory focus and the gain-loss frames on intertemporal decision making were examined. The results showed that the main effect of the gain-loss frames was significant, *F*(1, 49) = 7.91, *p* = 0.007, *p* < 0.05, *η*_p_^2^ = 0.14, the delay discounting rate was smaller in the loss frame (*M* = −5.78, *SD* = 0.35) than in the gain frame (*M* = −5.12, *SD* = 0.27). The main effects of chronic regulatory focus was not significant, *F*(1, 49) = 1.63, *p* = 0.208, *p* > 0.05, *η*_p_^2^ = 0.03. The interaction of gain-loss frames and chronic regulatory focus was not significant, *F*(1, 49) = 0.45, *p* = 0.505, *p* > 0.05, *η*_p_^2^ = 0.01. Further analysis about the differences between gain-loss frame among regulatory focus, results showed that for promotive focus participants, there was no significant difference in delay discounting rates between the loss frame (*M* = −5.33, *SD* = 2.55) and the gain frame (*M* = −4.83, *SD* = 2.03), *t*(25) = 1.56, *p* = 0.132, *p >* 0.05, 95% CI = [−0.16, 1.18], Cohen’s *d* = 0.22. For preventive focus participants, delay discounting rates were smaller in the loss frame (*M* = −6.23, *SD* = 2.43) compared to that in the gain frame (*M* = −5.41, *SD* = 1.82), *t*(24) = 2.39, *p* = 0.025, *p <* 0.05, 95% CI = [0.11, 1.54], Cohen’s *d* = 0.38. And results about the differences between promotive and preventive focus among gain-loss frame showed that, in the gain frame, there was no significant difference in delay discounting rates between promotive focus (*M* = −4.82, *SD* = 2.03) and preventive focus (*M* = −5.41, *SD* = 1.82), *t*(49) = 1.07, *p =* 0.290, *p >* 0.05, 95% CI = [−0.51, 1.67], Cohen’s *d* = 0.31. In the loss frame, there was no significant difference in delay discounting rates between promotive focus (*M* = −6.10, *SD* = 1.63) and preventive focus (*M* = −6.30, *SD* = 1.61), *t*(49) = 1.28, *p =* 0.0205, *p >* 0.05, 95% CI = [−0.51, 2.30], Cohen’s *d* = 0.36, see Figure 3.

### 3.6. Experiment 2 results

Experiment 2 found that in the upward social comparison condition, preventive focus participants under the loss frame were more inclined to choose later-smaller losses (i.e., later-larger benefit) than promotive focus participants. There was no significant difference between promotive focus and preventive focus participants in the intertemporal decision making under the gain frame. And, in the upward social comparison condition, promotive focus participants were more inclined to choose later-larger benefit under the gain frame than under the loss frame. Preventive focus participants were more inclined to choose later-smaller losses (i.e., later-larger benefit) in the intertemporal decision making under the loss frame than under the gain frame. While in the downward social comparison, there was no significant difference in intertemporal decision making between gain frame and loss frame for either promotive focus or preventive focus participants. This suggests that upward social comparison strengthened the effect of chronic regulatory focus on intertemporal decision making in the gain-loss frames, while downward comparison attenuated the effect, which was consistent with hypothesis 2.

## 4. Discussion

The present study aimed to explore the effect of social comparison and chronic regulatory focus on undergraduates’ intertemporal decision making in the gain-loss frames. Experiment 1 found that chronic regulatory focus influenced intertemporal decision making in the gain-loss frames, with preventive focus participants preferring later-smaller losses, that is later-larger gains, in the loss frame than in the gain frame. In contrast, individuals with promotive focus did not differ in the gain-loss frames. From another perspective of results, in the gain frame, participants with promotive focus were more inclined to choose LL than participants with preventive focus. In the loss frame, participants with preventive focus and promotive focus did not show significant difference in intertemporal choices. Experiment 2 found that social comparison moderated the effect of chronic regulatory focus on intertemporal decision making in the gain-loss frames. Specifically, upward social comparison enhanced the effect of chronic regulatory focus on intertemporal decision making in the gain-loss frames, whereas downward social comparison attenuated this effect.

According to the results of experiment 1, in gain frame, promotive focus individuals were more inclined to choose LL choices than preventive focus individuals. This conclusion was consistent with previous studies that promotive focus individuals have a higher sensitivity in the gains (Zou et al., 2014), so they tend to choose LL choice to get great gains. However, there was no significant differences between promotive focus and preventive focus individuals’ intertemporal decisions in loss frame. Earlier studies mentioned that for equal amounts of gain and loss, the effect of loss was greater than the choice in gain (Kahneman and Tversky, 1979). Thus, it was possible that promotive focus individuals tend to choose to avoid bigger loss in loss frame.

From another perspective of results in experiment 1, for preventive focus individuals, delay discount rate of intertemporal decisions in loss frame was smaller than that in gain frame. Previous researches have shown that preventive focus individuals were more sensitive to losses (Lin and Wang, 2007; Yao and Yue, 2009), and took a longer-term horizon in intertemporal decision making to avoid loss (Duclos and Khamitov, 2019), were able to predict consequences of decisions made in the loss context (Scholer et al., 2010). Preventive focus individuals, therefore, will make more long-term choices in the loss frame (later-smaller losses choices) than in the gain frame (later-larger rewards choices), due to the sensitivity of losses. While, for promotive focus individuals, there was no significant difference of intertemporal decisions in gain and loss frame. As mentioned before, due to the greater effect of loss, promotive focus individuals might choose LL in loss frame (Kahneman and Tversky, 1979).

According to the results of experiment 2, when upward comparing, in loss frame, individuals with preventive focus preferred LL choice than promotive focus. That is to say, preventive focus individuals were sensitive to losses, which was the replication of experiment 1 and in line with previous studies. While there is no significant difference between promotive focus and preventive focus in gain frame, which was inconsistent with the hypothesis and previous studies. Based on social comparison theory, when individuals compare themselves with others who are better than them, individuals will feel self-threatened (Han and Chi, 2012) and try to take steps to narrow the gap between themselves and others (Suls and Wheeler, 2000). While such comparison did not make promotive focus individuals prefer LL choice, it might be the reason that self-threatened, induced by upward comparison, make promotive focus individual less likely to choose LL choice in gain frames.

From another perspective of results in experiment 2, when upward comparing, for preventive focus undergraduates, the delay discount rate was significantly smaller in loss frame than that in gain frame, which represents the sensitivity to loss. This was consistent with our hypothesis and previous studies. While, to our surprise, the conclusion remained the same for promotive focus individuals which was contrary with previous studies. According to Xie and Lu (2014), upward social comparison leads to social losses, downward social comparison leads to social gains. Individuals might be more easily influenced by negative information when under a situation of social loss, causing stronger negative feelings and the appearance of loss aversion behaviors (Wang et al., 2016). The study suggests that upward social comparisons in loss frame might imply “double loss,” which may result in participants’ vigilance against double loss (Hu et al., 2021). Thus, promotive focus individuals tend to avoid bigger losses in loss frame; preventive focus individuals, who are more sensitive to losses and in a worse position during upward social comparison, will take all necessary actions to avoid losses in order to transfer their inferior position to a better one as much as possible (Scholer et al., 2010). As a result, upward social comparison seems enhanced the effect of chronic regulatory focus on intertemporal decision making in the gain-loss frames, that compared to experiment 1.

In downward social comparison, people perceive themselves as currently superior to others, and experience self-satisfaction and less stress (Xing and Yu, 2006). Although previous researches confirmed that individuals with promotive focus are more sensitive to gain and individuals with preventive focus are more sensitive to loss. But, they do not experience such ego-threat as in upward social comparison. This experience of success compared with others makes the promotive focus and preventive focus individuals have no strong desire to change current status. In the gain frame, the motivation of promotive focus individuals to achieve greater benefits is relatively weakened. Similarly, the incentive for preventive focus individuals to avoid greater losses is also relatively weakened in the loss frame. As a result, there is no significant difference between the intertemporal decisions of promotive focus and preventive focus individuals in the gain-loss frames when downward comparing.

According to the regulatory focus theory, both promotive focus and preventive focus are independent of negative and positive motivation. For example, the motivation to approach a positive goal state can be either promotion focus (i.e., focus on gain) and prevention focus (i.e., focus on no loss); similarly, for avoiding negative goals (i.e., focus on no loss; Molden et al., 2008). It has been shown that regulatory focus was positively related to behavioral activation-reward (BAS-R) and behavioral activation-pleasure seeking (BAS-F). And behavioral inhibition system was positively related to prevention focus (Lanaj et al., 2012). Previous results showed that people with a higher BIS score tended to choose LL rewards (Zhao et al., 2015) and BAS is marginally related to choice preference on SS rewards, and people with higher BAS scores tended to prefer a SS reward over a LL reward when they were in a temporarily angry mood (Zhao et al., 2017). There are currently no relevant studies on intertemporal decision making in the gain-loss framework for BAS and BIS, which could be studied further in future studies.

## 5. Theoretical implications

Previous research have found that preventive focus individuals were more concerned about taking necessary measures to avoid losses, whereas promotive focus individuals were more concerned about obtaining gains (Brockner and Higgins, 2001; Brockner et al., 2002). Few studies investigated how chronic regulatory focus affected intertemporal decision making in the gain-loss frames. This study examined the impact of chronic regulatory focus on intertemporal decision making in the gain-loss frames by providing a new theoretical perspective of social comparison to intertemporal decision researches.

In recent years, the problems concerning undergraduates’ money choices have become more and more prominent, such as the problem of borrowing, living expenses over value, etc. Behind these problems, it shows that there are certain problems of delayed satisfaction among undergraduates. The present study also had important practical implications. The results might provide suggestions for undergraduates to make farsighted decisions, such as, presenting the choices in the form of loss and comparing with superior others, which may prevent undergraduates from making short-sighted choices. And under the upward social comparison, it will make the college students who promotive focus become long-term in the loss frame, and the college students who prevention focus are more able to avoid losses under the loss frame.

## 6. Limitations and future research

This study also has some limitations. It worth noting that upward social comparison may motivate individuals’ self-improvement, which in turn influences intertemporal decision making in the gain-loss frames. However, this study did not examine the mediating effect of self-improvement directly, which need to be examined further in the future research. Second, this study is a laboratory experiment, the designs might raise concerns about how spontaneous social comparisons among college students with different regulatory focus influence intertemporal decision making in realistic scenarios based on real consumption and other decision making situations, which future researches may take efforts to explore.

## 7. Conclusion

Chronic regulatory focus influenced intertemporal decision making in the gain-loss frames, with preventive focus undergraduates preferring small, but later losses, that is large, long-term gains, in the loss frame than in the gain frame. In contrast, individuals with promotive focus did not differ in the gain-loss frames. Upward social comparison elevated the effect of chronic regulatory focus on intertemporal decision making in the gain-loss frames. Compared to the downward social comparison, under upward social comparison, both promotive focus undergraduates and preventive focus undergraduates tend to be more visionary in loss frame. And under upward social comparison, the extent to which preventive focus undergraduates were more farsighted than promotive focus undergraduates in the loss domain became larger.

## Data availability statement

The raw data supporting the conclusions of this article will be made available by the authors, without undue reservation.

## Ethics statement

The studies involving human participants were reviewed and approved by Ethics Committee of Ludong University. The patients/participants provided their written informed consent to participate in this study.

## Author contributions

DL designed the experiment, analyzed experiment data and wrote the original manuscript. YZ reviewed and edited the manuscript. XG supervised the work. All authors have read and agreed to the published version of the manuscript.

## Funding

This research was partially supported by the National Natural Science Foundation of China (71971104), the Ministry of Education of Humanities and Social Science project (19YJA190002), and Science and Technology Support Plan for Youth Innovation of Universities in Shandong Province (2019RWF001).

## Conflict of interest

The authors declare that the research was conducted in the absence of any commercial or financial relationships that could be construed as a potential conflict of interest.

## Publisher’s note

All claims expressed in this article are solely those of the authors and do not necessarily represent those of their affiliated organizations, or those of the publisher, the editors and the reviewers. Any product that may be evaluated in this article, or claim that may be made by its manufacturer, is not guaranteed or endorsed by the publisher.

## References

[ref1] Brockner J. Higgins E. T. (2001). Regulatory focus theory: implications for the study of emotions at work. Organ. Behav. Hum. Decis. Process. 86, 35–66. doi: 10.1006/obhd.2001.2972

[ref2] Brockner J. Paruchuri S. Idson L. C. Higgins E. T. (2002). Regulatory focus and the probability estimates of conjunctive and disjunctive events. Organ. Behav. Hum. Decis. Process. 87, 5–24. doi: 10.1006/obhd.2000.2938

[ref3] Calluso C. Tosoni A. Fortunato G. Committeri G. (2017). Can you change my preferences? Effect of social influence on intertemporal choice behavior. Behav. Brain Res. 330, 78–84. doi: 10.1016/j.bbr.2017.05.001, PMID: 28478066

[ref4] Chen J. X. He G. B. (2015). Measuring the temporal discounting of environmental outcomes: comparison between matching method and titration on procedure. Chin. J. Appl. Psychol. 21, 12–20. doi: 10.3969/j.issn.1006-6020.2015.01.002

[ref5] David J. H. Katherine F. T. David H. K. Elke U. W. (2013). How to measure time preferences: an experimental comparison of three methods. Judgm. Decis. Mak. 8, 236–249.

[ref6] Dhandra T. K. (2020). Does self-esteem matter? A framework depicting role of self-esteem between dispositional mindfulness and impulsive buying. J. Retail. Consum. Serv. 55:102135. doi: 10.1016/j.jretconser.2020.102135

[ref7] Dou W. Qu L. L. Qu C. (2014). Social comparison affects outcome evaluation in the cooperative task: an ERP study. Acta Psychol. Sin. 46, 405–414. doi: 10.3724/SP.J.1041.2014.00405

[ref8] Duclos R. Khamitov M. (2019). Compared to dematerialized money, cash increases impatience in intertemporal choice. J. Consum. Psychol. 29, 445–454. doi: 10.1002/jcpy.1098

[ref9] Faul F. Erdfelder E. Lang A. G. Buchner A. (2007). G*power 3: a flexible statistical power analysis program for the social, behavioral, and biomedical sciences. Behav. Res. Methods 39, 175–191. doi: 10.3758/bf03193146, PMID: 17695343

[ref10] Faralla V. Novarese M. Ardizzone A. (2017). Framing effects in intertemporal choice: a nudge experiment. J. Behav. Exp. Econ. 71, 13–25. doi: 10.1016/j.socec.2017.09.002

[ref11] Festinger L. (1954). A theory of social comparison processes. Hum. Relat. 7, 117–140. doi: 10.1177/001872675400700202

[ref12] Frederick S. Loewenstein G. O'Donoghue T. (2002). Time discounting and time preference: a critical review. J. Econ. Lit. 40, 351–401. doi: 10.1257/002205102320161311

[ref13] Gehring W. J. Willoughby A. R. (2002). The medial frontal cortex and the rapid processing of monetary gains and losses. Science 295, 2279–2282. doi: 10.1126/science.1066893, PMID: 11910116

[ref14] Gilman J. M. Curran M. T. Calderon V. Stoeckel L. E. Evins A. E. (2014). Impulsive social influence increases impulsive choices on a temporal discounting task in young adults. PLoS One 9:e101570-8. doi: 10.1371/journal.pone.0101570, PMID: 24988440 PMC4079280

[ref15] Gong X. S. Zhang H. H. (2020). Outstanding others vs. mediocre me: the effect of social comparison on uniqueness-seeking behavior. Acta Psychol. Sin. 52, 645–658. doi: 10.3724/SP.J.1041.2020.00645

[ref16] Guo S. B. Huang X. T. (2010). Motive forces behind social comparison: motivations and orientation. J. Southwest Univ. 36, 14–18. doi: 10.13718/j.cnki.xdsk.2010.04.030

[ref17] Han X. Y. Chi Y. K. (2012). The self-threat of unsolicited social comparison and its balance. Acta Psychol. Sin. 44, 1628–1640. doi: 10.3724/SP.J.1041.2012.01628

[ref18] Higgins E. T. (1997). Beyond pleasure and pain. Am. Psychol. 52, 1280–1300. doi: 10.1037/0003-066X.52.12.1280 9414606

[ref19] Hu Y. X. Zhou M. M. Shao Y. R. Wei J. Li Z. Y. Xu S. K. (2021). The effects of social comparison and depressive mood on adolescent social decision-making. BMC Psychiatry 21, 3–15. doi: 10.1186/s12888-020-02928-y, PMID: 33402153 PMC7786518

[ref20] Kahneman D. Tversky A. (1979). Prospect theory: an analysis of decision under risk. Econometrica 47, 263–292. doi: 10.1017/cbo9780511609220.014

[ref21] Kirby K. N. Petry N. M. Bickel W. K. (1999). Heroin addicts have higher discount rates for delayed rewards than non-drug-using controls. J. Exp. Psychol. Gen. 128, 78–87. doi: 10.1037/0096-3445.128.1.78, PMID: 10100392

[ref22] Kirby K. N. (2009). One-year temporal stability of delay-discount rates. Psychon. Bull. Rev. 16, 457–462. doi: 10.3758/pbr.16.3.457, PMID: 19451368

[ref23] Lanaj K. Chang C. H. Johnson R. E. (2012). Regulatory focus and work-related outcomes: a review and meta-analysis. Psychol. Bull. 138, 998–1034. doi: 10.1037/a0027723, PMID: 22468880

[ref24] Lian S. L. Sun X. J. Niu G. F. Zhou Z. K. (2017). Upward social comparison on SNS and depression: a moderated mediation model and gender difference. Acta Psychol. Sin. 49, 941–952. doi: 10.3724/SP.J.1041.2017.00941

[ref25] Lin H. Y. Wang L. (2007). The regulatory fit theory. Adv. Psychol. Study 15, 749–753.

[ref26] Liu P. He J. L. Li A. (2019). Upward social comparison on social network sites and impulse buying: a moderated mediation model of negative affect and rumination. Comput. Hum. Behav. 96, 133–140. doi: 10.1016/j.chb.2019.02.003

[ref27] Lockwood P. Kunda Z. (1997). Superstars and me: predicting the impact of role models on the self. J. Pers. Soc. Psychol. 73, 91–103. doi: 10.1037/0022-3514.73.1.91

[ref28] Ma W. J. Su T. Li Y. D. Luo L. Z. Feng T. Y. Li H. (2012). Dissecting the win-loss framing effect of intertemporal choice: researches from intertemporal choice of money-gain & loss. Acta Psychol. Sin. 44, 1038–1046. doi: 10.3724/SP.J.1041.2012.01038

[ref29] Molden D. C. Lee A. Y. Higgins E. T. (2008). Motivations for promotion and prevention. Handbook of motivation science. Available at: books.google.com.

[ref30] Novak T. P. Hoffman D. L. (2009). The fit of thinking style and situation: new measures of situation-specific experiential and rational cognition. J. Consum. Res. 36, 56–72. doi: 10.1086/596026

[ref31] Pettit N. C. Lount R. B. Jr. (2010). Looking down and ramping up: the impact of status differences on effort in intergroup contexts. J. Exp. Soc. Psychol. 46, 9–20. doi: 10.1016/j.jesp.2009.08.008

[ref32] Pham M. T. Higgins E. T. (2005). “Promotion and prevention in consumer decision making: the state of the art and theoretical propositions,” in Inside Consumption: Consumer Motives, Goals, and Desires. eds. Ratneshwar S. Mick D. G. (London, UK: Routledge), 8–43.

[ref33] Ren T. H. Hu Z. S. Sun H. Y. Liu Y. Li S. (2015). Making a decision vs. sticking to a decision: a comparison of Intertemporal choice and delay of gratification. Adv. Psychol. Study 23, 303–315. doi: 10.3724/SP.J.1042.2015.00303

[ref34] Scholer A. A. Zou X. Fujita K. Stroessner S. J. Higgins E. T. (2010). When risk seeking becomes a motivational necessity. J. Pers. Soc. Psychol. 99, 215–231. doi: 10.1037/a0019715, PMID: 20658840

[ref35] Suls J. M. Wheeler L. (2000). Handbook of Social Comparison: Theory and Research. New York: Plenum Press.

[ref36] Thaler R. (1981). Some empirical evidence on dynamic inconsistency. Econ. Lett. 8, 201–207. doi: 10.1016/0165-1765(81)90067-7

[ref37] Tversky A. Kahneman D. (1981). The framing of decisions and the psychology of choice. Science 211, 453–458. doi: 10.1126/science.7455683 7455683

[ref38] Vogel E. A. Rose J. P. Aspiras O. G. Edmonds K. A. Gallinari E. F. (2020). Comparing comparisons: assimilation and contrast processes and outcomes following social and temporal comparison. Self Identity 19, 629–649. doi: 10.1080/15298868.2019.1647278

[ref39] Wang D. W. Hao L. L. Zhou M. M. Maguire P. Zhang X. Y. Zhang X. (2019). Making decisions for oneself and others: how regulatory focus influences the ‘decision maker role effect’ for intertemporal choices. Pers. Individ. Differ. 149, 223–230. doi: 10.1016/j.paid.2019.05.034

[ref40] Wang D. W. Zhu L. P. Maguire P. Liu Y. X. Pang K. Y. Li Z. Y. (2016). The influence of social comparison and peer group size on risky decision-making. Front. Psychol. 7:1232. doi: 10.3389/fpsyg.2016.01232, PMID: 27582723 PMC4987381

[ref41] Wang L. Lin H. Y. Pang X. M. (2011). The coincidence between the regulatory fit effects based on chronic regulatory focus and situational regulatory focus. Acta Psychol. Sin. 43, 553–560. doi: 10.3724/SP.J.1041.2011.00553

[ref42] Werth L. Foerster J. (2007). How regulatory focus influences consumer behavior. Eur. J. Soc. Psychol. 37, 33–51. doi: 10.1002/ejsp.343

[ref43] Wills T. A. (1981). Downward comparison principles in social psychology. Psychol. Bull. 90, 245–271. doi: 10.1037/0033-2909.90.2.245

[ref44] Wu P. Xiao L. Jiang Q. P. Zhang J. M. (2016). The win-loss framing effect of intertemporal choice: the role of timing and risk. Psychol. Explo Ration 36, 508–513.

[ref45] Xie X. F. Lu J. Y. (2014). Double reference points in risky decision making. Adv. Psychol. Sci. 22, 571–579. doi: 10.3724/SP.J.1042.2014.00571

[ref46] Xing S. F. Yu G. L. (2006). Social comparison: contrast effect or assimilation effect? Adv. Psychol. Study 14, 944–949.

[ref47] Xu L. J. Liang Z. Y. Wang K. Li S. Jiang T. Z. (2009). Neural mechanism of intertemporal choice: from discounting future gains to future losses. Brain Res. 1261, 65–74. doi: 10.1016/j.brainres.2008.12.061, PMID: 19185567

[ref48] Yao L. Liu F. H. Li W. Q. Zhang X. Q. Yuan B. (2022). The effect of competence social comparison and attributive feedback on dishonest behavior. Psychol. Dev. Educ. 38, 778–785. doi: 10.16187/j.cnki.issn1001-4918

[ref49] Yao Q. Yue G. A. (2009). New development in the domain of motivation: regulatory focus theory. Adv. Psychol. Study 17, 1264–1273.

[ref50] Yao Q. Yue G. A. Wu C. C. Li Y. F. Chen C. (2005). Measurement of regulatory focus: the reliability and validity of Chinese version of regulatory focus questionnaire. Chin. J. Appl. Psychol. 14, 318–323. doi: 10.3969/j.issn.1006-6020.2008.04.005

[ref51] Zhang X. (2018). Intertemporal Choice in Gain and Loss Situation: Evidence from Behavioral Experiments and ERP Abstract. Jinan, China: Shandong Normal University.

[ref52] Zhao J. L. Cheng J. Q. Harris M. Vigo R. (2015). Anxiety and intertemporal decision making: the effect of the behavioral inhibition system and the moderation effects of trait anxiety on both state anxiety and socioeconomic status. Pers. Individ. Differ. 87, 236–241. doi: 10.1016/j.paid.2015.08.018

[ref53] Zhao J. L. Kirwen N. Johnson J. Vigo R. (2017). Anger and intertemporal choice: the behavioral approach system and the interactive effects of trait and state anger. Pers. Individ. Differ. 110, 60–64. doi: 10.1016/j.paid.2017.01.022

[ref54] Zheng X. Y. Peng S. Q. Peng L. L. (2015). Feeling better and becoming MoreBenevolent: impact of social comparison on prosocial behavior. Acta Psychol. Sin. 47, 243–250. doi: 10.3724/SP.J.1041.2015.00243

[ref55] Zhou R. R. Zhao S. H. (2009). Motivational influences in time discounting: the effect of regulatory focus. Regul. Focus Discount. 1–43. doi: 10.2139/ssrn.2333362

[ref56] Zilker V. Pachur T. (2021). Does option complexity contribute to the framing effect, loss aversion, and delay discounting in younger and older adults? J. Behav. Decis. Mak. 34, 488–503. doi: 10.1002/bdm.2224

[ref57] Zou X. Scholer A. A. Higgins E. T. (2014). In pursuit of progress: promotion motivation and risk preference in the domain of gains. J. Pers. Soc. Psychol. 106, 183–201. doi: 10.1037/a0035391, PMID: 24467419

